# PHLPP negatively regulates cell motility through inhibition of Akt activity and integrin expression in pancreatic cancer cells

**DOI:** 10.18632/oncotarget.6848

**Published:** 2016-01-08

**Authors:** Alena J. Smith, Yang-An Wen, Payton D. Stevens, Jingpeng Liu, Chi Wang, Tianyan Gao

**Affiliations:** ^1^ Markey Cancer Center, University of Kentucky, Lexington, KY, USA; ^2^ Department of Molecular and Cellular Biochemistry, University of Kentucky, Lexington, KY, USA

**Keywords:** pancreatic cancer, cell migration, tumor suppressor, PHLPP, integrin

## Abstract

Pancreatic adenocarcinoma is currently the fourth leading cause for cancer-related mortality. Malignant progression of pancreatic cancer depends not only on rapid proliferation of tumor cells but also on increased cell motility. In this study, we showed that increased PHLPP expression significantly reduced the rate of migration in pancreatic ductal adenocarcinoma (PDAC) cells whereas knockdown of PHLPP had the opposite effect. In addition, cell motility at the individual cell level was negatively regulated by PHLPP as determined using time-lapse imaging. Interestingly, the expression of β1 and β4 integrin proteins were decreased in PHLPP overexpressing cells and increased in PHLPP knockdown cells whereas the mRNA levels of integrin were not altered by changes in PHLPP expression. In determining the molecular mechanism underlying PHLPP-mediated regulation of integrin expression, we found that inhibition of lysosome activity rescued integrin expression in PHLPP overexpressing cells, thus suggesting that PHLPP negatively controls cell motility by inhibiting Akt activity to promote lysosome-dependent degradation of integrins. Functionally, the increased cell migration observed in PHLPP knockdown cells was effectively blocked by the neutralizing antibodies against β1 or β4 integrin. Taken together, our study identified a tumor suppressor role of PHLPP in suppressing cell motility by negatively regulating integrin expression in pancreatic cancer cells.

## INTRODUCTION

Pancreatic adenocarcinoma is the fourth leading cause of cancer related deaths in the United States, despite being the ninth most diagnosed cancer in men and the eleventh in women. PDAC arises from the exocrine portion of the pancreas and accounts for 95% of all pancreatic adenocarcinoma [1]. PDAC has a dismal 5-year survival rate of 4% and a median survival span of 6 months from the point of diagnosis, and it has a high rate of chemotherapy and radiation resistance [1]. Thus, a better understanding of the molecular events leading to cancer progression is needed in order to improve the treatment and prognosis of PDAC patients.

Accumulation of genetic mutations in oncogenes and tumor suppressors lead to the development of pre-malignant lesions which eventually through genetic instability give rise to carcinoma within the pancreatic ductal epithelium [2]. Key mutations in oncogenes such as *KRAS* with deactivation of tumor suppressor genes *SMAD4* and *CDKN2A/INK4A* have been implicated in the development and progression of pancreatic cancer [3, 4]. In addition, it has been shown that overexpression of integrin α6β4 promotes migration and invasion of pancreatic cancer cells and is associated with the progression of PDAC [5, 6]. Integrins are known to contribute to tumor progression and metastasis by directly activating a number of oncogenic signaling pathways, including PI3K/Akt and RAS/RAF pathways, in various types of cancer [7, 8]. However, the molecular mechanism by which the expression of integrin proteins is regulated remains elusive in pancreatic cancer cells.

PHLPP (PH domain leucine-rich repeat protein phosphatase) belongs to a novel family of Ser/Thr protein phosphatases. There are two isoforms, PHLPP1 and PHLPP2, identified in this family [9–12]. Both PHLPP isoforms were first discovered as the phosphatases for Akt that directly dephosphorylate the hydrophobic motif Ser473 site and inactivate the kinase [9, 10]. Moreover, it has been shown that PHLPP dephosphorylates Ser338, a key activation site on RAF1, and inhibits the downstream signaling through RAF/MEK/ERK in colon cancer cells [13]. Therefore, PHLPP may exert its tumor suppressor function by negatively regulating both the PI3K/Akt and RAS/RAF pathways. Recently, Nitsche et al discovered that there is a stage-dependent downregulation of PHLPP in pancreatic cancer patient specimens, thus suggesting a tumor suppressor role of PHLPP in pancreatic cancer [14]. However, it remains unknown whether loss of PHLPP expression promotes cancer cell migration in pancreatic cancer.

In this study, we determined the role of PHLPP in regulating cell migration and motility in pancreatic cancer cells. We identified a functional connection between PHLPP expression and integrin function. Results from our study revealed that PHLPP-loss increases cell motility by upregulating integrin expression and inducing EMT. Furthermore, we found that PHLPP negatively controls integrin expression by promoting lysosome-mediated degradation of integrin via inhibition of Akt.

## RESULTS

### PHLPP negatively regulates the activity of Akt and MEK/ERK in pancreatic cells

**Figure 1:**
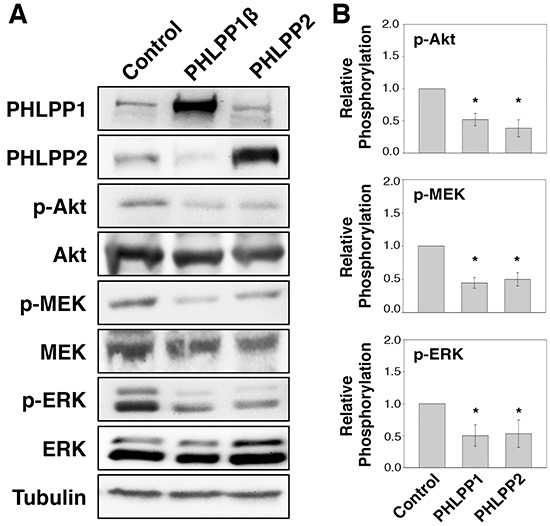
Overexpression of PHLPP isoforms inhibit PI3K/Akt and MEK/ERK signaling **A.** Stable control, HA-PHLPP1β or HA-PHLPP2 overexpressing Panc-1 cells were generated using retrovirus-mediated infection. The cell lysates were prepared and analyzed for phosphorylation and total protein expression by immunoblotting. **B.** Relative phosphorylation for p-Akt, p-MEK, p-ERK were calculated and normalized to those of total Akt, MEK and ERK, respectively. The level in control cells was set to 1. Data represent the mean ± SEM (*n* = 3, * *p*<0.05 by two-sample *t*-tests).

**Figure 2:**
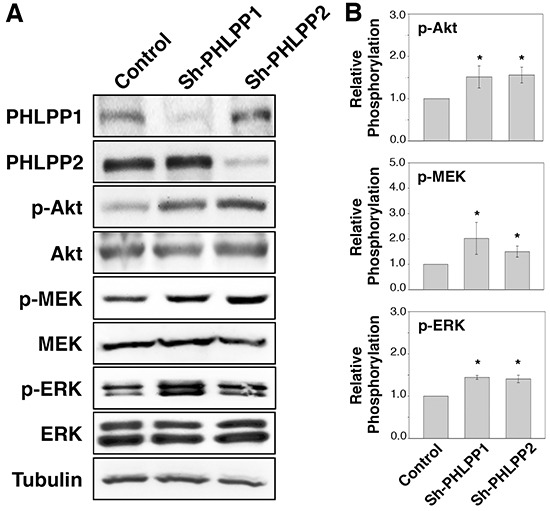
Loss of PHLPP expression enhances PI3K/Akt and MEK/ERK signaling **A.** Stable control (sh-Con) and PHLPP knockdown (sh-PHLPP1 and sh-PHLPP2) ASPC-1 cells were generated using lentivirus-mediated RNAi. The cell lysates were analyzed for phosphorylation and total protein expression by immunoblotting. **B.** Relative phosphorylation for p-Akt, p-MEK, p-ERK were calculated and normalized to those of total Akt, MEK and ERK, respectively. The level in control cells was set to 1. Data represent the mean ± SEM (*n* = 3, * *p*<0.05 by two-sample *t*-tests).

To determine if PHLPP serves as a tumor suppressor in human pancreatic cancer, we established stable cell lines overexpressing PHLPP1 or PHLPP2 in Panc-1 cells, which express very low levels of endogenous PHLPPs. The PHLPP1 gene potentially encodes two differentially spliced variants, PHLPP1α and PHLPP1β [11]. Since the longer transcript of PHLPP1, PHLPP1β, is the predominant form expressed endogenously in all pancreatic cell lines examined, we used PHLPP1β in our study. We first determined the effect of PHLPP overexpression on cell signaling. As shown in Figure 1, both Akt and MEK/ERK activity were downregulated in PHLPP overexpressing cells compared to control cells as indicated by decreased phosphorylation of Akt, MEK, and ERK. Next, to determine the effect of endogenous PHLPP on Akt and MEK/ERK signaling, PHLPP was silenced in ASPC-1 cells, which express relatively higher levels of endogenous PHLPPs, using lentiviral-mediated RNAi. Immunoblotting results revealed that phosphorylation of Akt, MEK, and ERK was significantly elevated when PHLPP expression was knocked down (Figure 2). Consistent with previous reports on the tumor suppressor function of PHLPP in other cancer types [13, 15–18], our results here provide the initial evidence that PHLPP is capable of inhibiting both Akt and MEK/ERK signaling in pancreatic cancer cells.

### PHLPP negatively regulates cell migration in pancreatic cancer cells

**Figure 3:**
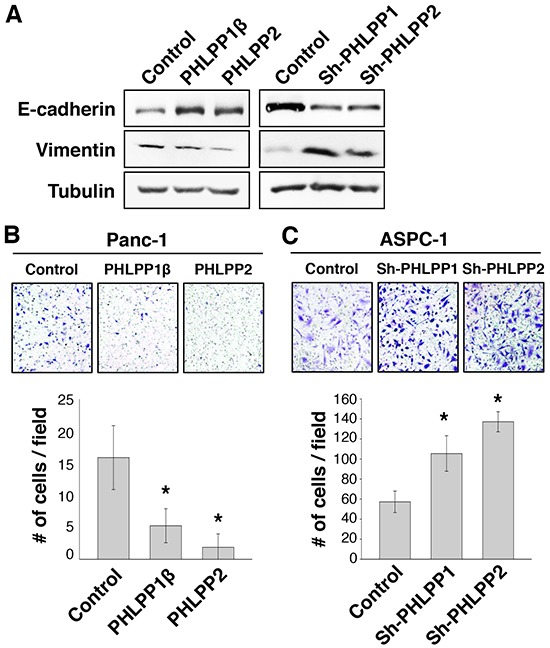
Knockdown of PHLPP expression induces EMT and promotes cell migration **A.** Cell lysates were prepared from stable control and PHLPP overexpressing Panc-1 as well as PHLPP knockdown ASPC-1 cells. The expression levels of E-cadherin and vimentin were analyzed using immunoblotting. **B.** Stable control and PHLPP overexpressing Panc-1 cells were subjected to Transwell migration assays using HGF and laminin as chemoattractants. **C.** Stable control and PHLPP knockdown ASPC-1 cells were subjected to Transwell migration assays using HGF and laminin as chemoattractants. The inserts shown in (B) and (C) were representative images of cells migrated in the Transwell assays. Each experiment was done in duplicates and three independent experiments were averaged and expressed as mean ± SEM (* *p*<0.05 by two-sample *t*-tests compared to the control cells).

Previous studies of PHLPP expression in colorectal cancer cells showed an enhanced ability for PHLPP knockdown cells to migrate and transition into EMT [13]. To test if PHLPP regulates cell motility in pancreatic cancer cells, we first examined the expression of EMT markers in PHLPP overexpressing Panc-1 and PHLPP knockdown ASPC-1 cells. As shown in Figure 3A, PHLPP overexpression markedly increased the expression of E-cadherin, an epithelial cell marker, and decreased the expression of vimentin, a mesenchymal cell marker. Conversely, knockdown of either PHLPP isoform decreased E-cadherin whereas increased vimentin expression (Figure 3A), suggesting that PHLPP downregualtion promotes EMT in pancreatic cancer cells. Furthermore, we found that PHLPP overexpressing cells migrated significantly slower than the control cells as determined by Transwell migration assays (Figure 3B). In contrast, knockdown of PHLPP significantly enhanced the ability of pancreatic cancer cells to migrate in response to HGF. Similar results were obtained when using IGF-1 and collagen were used as chemoattractants (Supplementary Figure S1).

**Figure 4:**
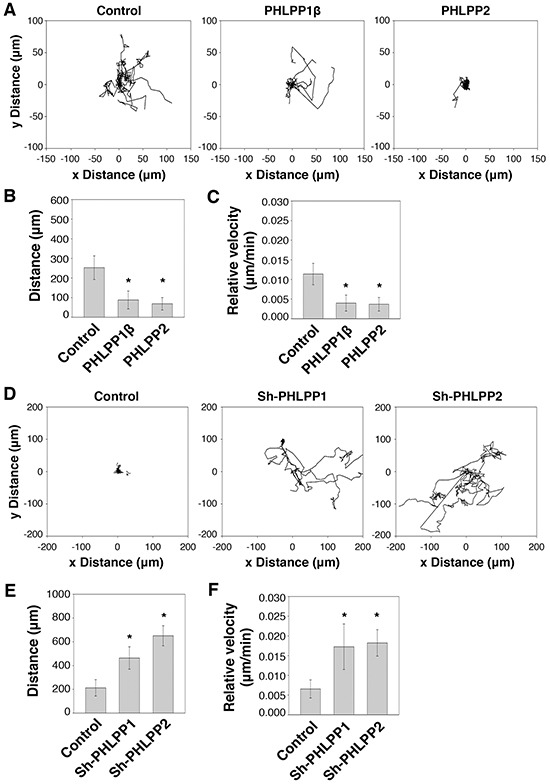
PHLPP controls cell motility at the single cell level **A.** Migration patterns of stable control and PHLPP overexpressing Panc-1 cells on laminin coated plates using HGF as the chemoattractant. The trajectories of 12 randomly chosen cells for each cell line were plotted in the graphs. **B–C.** The average distance traveled (B) and the relative velocity (C) of 12 stable control and PHLPP overexpressing Panc-1 cells during 6 hours of migration. Data represent mean ± SD (*n* = 12 cells/line, * *p*<0.05 by two-sample *t*-tests). **D.** Migration patterns of stable control and PHLPP knockdown ASPC-1 cells on laminin coated plates using HGF as the chemoattractant. The trajectories of 12 randomly chosen cells for each cell line were plotted in the graphs. **E–F.** The average distance traveled (E) and the relative velocity (F) of 12 stable control and PHLPP knockdown ASPC-1 cells during 6 hours of migration are shown. Data represent mean ± SEM (*n* = 12 cells/line, * *p*<0.05 by two-sample *t*-tests).

We next determined the effect of PHLPP on modulating cell motility at the single cell level. To this end, PHLPP overexpressing Panc-1 and PHLPP knockdown ASPC-1 cells were subjected to cell tracking experiments to monitor the cell movement in real time. We found that overexpression of PHLPP resulted in a significant decrease in cell motility when compared to the control cells (Figure 4A), whereas PHLPP knockdown cells were considerably more motile (Figure 4D). The velocity and average distances traveled were significantly decreased in PHLPP overexpressing cells (Figure 4B–4C) while significantly increased in PHLPP knockdown cells (Figure 4E–4F). Taken together, we have identified a role of PHLPP in inhibiting EMT and cell motility in pancreatic cancer cells.

### PHLPP inhibits the invasive growth of pancreatic cancer cells in 3D culture

**Figure 5:**
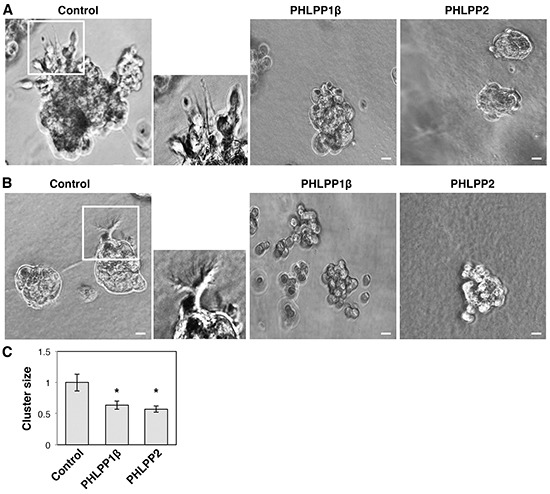
PHLPP inhibits the invasive phenotype of pancreatic cancer cells grown in 3D culture **A–B.** Stable control and PHLPP overexpressing Panc-1 cells were seeded in Matrigel and allowed to grow for 7 days. On the 3^rd^ day (A) and the 7^th^ day (B) after seeding phase-contrast images were taken with the 10X objective on using an inverted Nikon microscope. The boxed regions were enlarged and shown next to the original images. Scale bars, 100 μm. **C.** The size of cell clusters at day 7 was measured and analyzed using Nikon Element AR software. Data represent mean ± SEM (*n* = 18 cells/line, * *p*<0.05 by two-sample *t*-tests).

**Figure 6:**
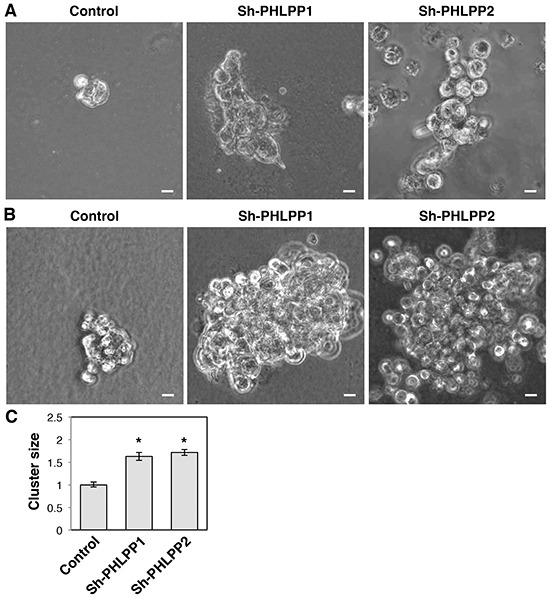
Knockdown of PHLPP promotes the invasive growth of pancreatic cancer cells in 3D culture **A–B.** Stable control and PHLPP knockdown ASPC-1 cells were seeded in Matrigel and allowed to grow for 7 days. On the 5^th^ day (A) and the 7^th^ day (B) after seeding phase-contrast images were taken with the 10X objective on using an inverted Nikon microscope. Scale bars, 100 μm. **C.** The size of cell clusters at day 7 was measured and analyzed using Nikon Element AR software. Data represent mean ± SEM (*n* = 18 cells/line, * *p*<0.05 by two-sample *t*-tests).

To further determine the effect of PHLPP on invasive growth of pancreatic cancer cells, we used 3D cell culture systems as they closely mimic the cancer microenvironment and they provide a better opportunity to explore the effects of oncogenes and tumor suppressors on modulating morphogenesis as well as invasive growth [19, 20]. Both PHLPP overexpressing Panc-1 and PHLPP knockdown ASPC-1 cells were seeded into Matrigel and allowed to grow for up to 7 days. The control Panc-1 cells grew into large multi-globular clusters with spikes of membrane protrusions extending into Matrigel (Figure 5A–5B). However, the formation of membrane protrusions was completed blocked in PHLPP overexpressing cells and the size of cell clusters was significantly decreased (Figure 5C). In addition, TIRF images of control Panc-1 cells showed the formation of both stress fibers and long filamentous extensions at the basolateral membrane surrounding the entire cell whereas very few actin stress fibers were observed in PHLPP overexpressing cells (Supplementary Figure S2). Although no clear membrane protrusions were observed in ASPC-1 cells grown in Matrigel, PHLPP knockdown cells formed significantly larger globular clusters compared to the control cells, suggesting that PHLPP-loss increases the invasive growth ability of pancreatic cancer cells (Figure 6A–6C).

### PHLPP regulates the expression of integrin in pancreatic cancer cells

**Figure 7:**
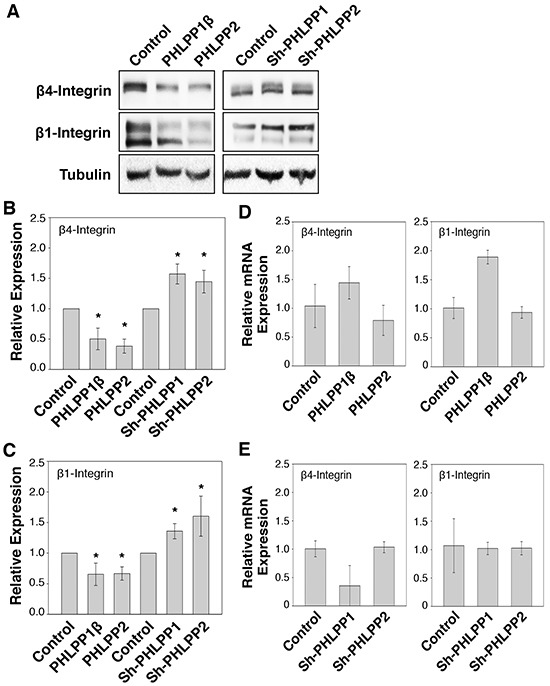
PHLPP regulates integrin protein expression **A.** Cell lysates prepared from PHLPP overexpressing Panc-1 and PHLPP knockdown ASPC-1 cells were analyzed for the expression of β1 and β4 integrin and tubulin using immunoblotting. **B–C.** Relative expression of β1 (B) and β4 integrin (C) was calculated and normalized to tubulin. The level in control cells was set to 1. Data represent the mean ± SEM (*n* = 3, * *p*<0.05 by two-sample *t*-tests). **D–E.** Total RNAs were isolated from PHLPP overexpressing Panc-1 cells (D) and PHLPP knockdown ASPC-1 cells (E). Real-time PCR analysis was performed using probes specific for human β1 and β4 integrin genes. Each experimental point was done in triplicates, and the graphs represent the mean ± SD (*n* = 3).

Previous studies have shown that increased β4 integrin expression is associated with pancreatic cancer progression [5]. Interestingly, when examining the expression of β integrin, we found that expression of both β4 and β1 integrin were significantly reduced in PHLPP overexpressing Panc-1 cells and increased in the PHLPP knockdown ASPC-1 cells (Figure 7A–7C). To further determine if PHLPP-mediated regulation of β4 and β1 integrin expression is controlled at the transcriptional level, total RNAs were isolated from PHLPP overexpressing Panc-1 and PHLPP knockdown ASPC-1 cells, and real-time PCR (RT-PCR) analysis was performed using probes specific for the β4 or β1 integrin gene. The results revealed that the mRNA levels of β4 and β1 integrin were not decreased by changes in PHLPP expression (Figure 7D–7E). Interestingly, the levels of β4 and β1 integrin mRNA were increased in PHLPP1β overexpressing cells. However, since the expression of β4 and β1 integrin proteins was decreased in PHLPP1β overexpressing cells, this increase of integrin mRNA was unlikely the reason for PHLPP-mediated downregulation of integrin. Collectively, these results indicated that PHLPP negatively regulates integrin expression via a post-translational mechanism in pancreatic cancer cells.

**Figure 8:**
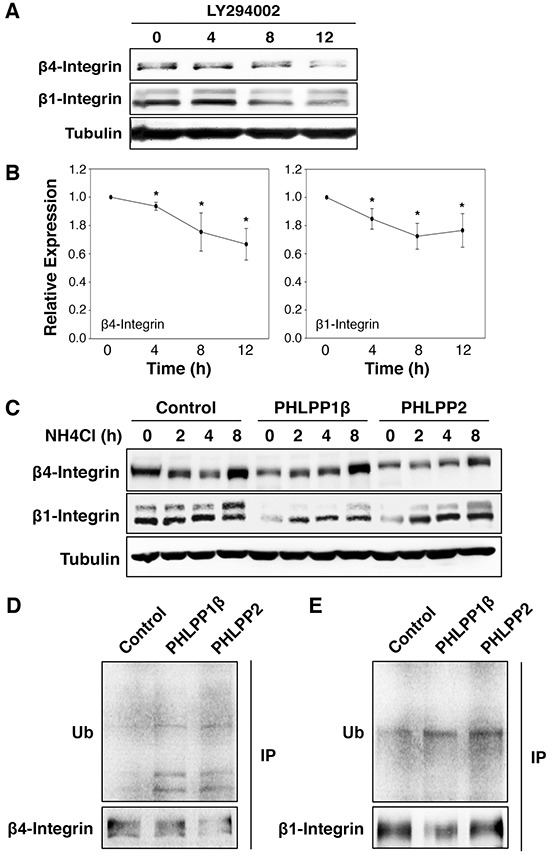
Inhibition of PI3K/Akt pathway promotes lysosome-mediated integrin degradation **A.** ASPC-1 cells were incubated with LY294002 for 0–12 hours. At indicated time points, cell lysates were prepared and analyzed for the expression of β1 and β4 integrin and tubulin using immunoblotting. **B.** Relative expression levels of β1 and β4 integrin at different time points following LY294002 treatment in ASPC-1 cells were calculated and normalized to tubulin. The level in untreated cells was set to 1. Data represent the mean ± SEM (*n* = 3, * *p*<0.05 by two-sample *t*-tests). **C.** Stable control and PHLPP overexpressing Panc-1 cells were treated with NH_4_Cl (5 mM) for 2, 4, and 8 hours. Cell lysates were prepared and analyzed by immunoblotting for integrin expression. **D.** Cell lysates prepared from stable control and PHLPP overexpressing Panc-1 cells were immunoprecipitated with antibodies against β1 or β4 integrin. The ubiquitination of endogenous β1 and β4 integrin was detected using the anti-ubiquitin antibody.

To further investigate the mechanism by which PHLPP regulates integrin expression, we tested if the PI3K/Akt or the MEK/ERK pathway is involved in controlling the expression of integrin proteins. Inhibiting the PI3K/Akt pathway using a PI3K inhibitor, LY294002, in ASPC-1 cells resulted in an approximately 40% reduction in both β4 and β1 integrin expression over time (Figure 8A–8B). A similar decrease of integrin expression was seen in cells treated with an Akt inhibitor, Akt-VIII (Supplementary Figure S3). However, treating cells with a MEK inhibitor, PD325901, had no effect on the expression of integrin (Supplementary Figure S3), suggesting that PHLPP-induced downregulation of integrin is likely mediated through inhibition of Akt signaling. As a control, we found that the mRNA levels of integrin were not significantly changed in cells treated with LY294002 (Supplementary Figure S3).

Recent studies have suggested that integrins can be internalized upon binding to extracellular matrix (ECM) ligands and trafficked to lysosome for degradation [21–24]. To determine if decreased integrin expression seen in PHLPP overexpressing cells is due to increased lysosomal degradation, we incubated control and PHLPP overexpressing Panc-1 cells in NH_4_Cl containing media. The presence of NH_4_Cl, a lysosomotropic alkalinizing agent, inhibits lysosomal protein degradation. At the end of 8-hour treatment with NH_4_Cl, both β4 and β1 integrin expression increased in response to lysosome inhibition and the expression of integrin in PHLPP overexpressing cells was similar to that of the control cells (Figure 8C). Similar results were obtained in cells treated with a different lysosome inhibitor, chloroquine (Supplementary Figure S3). In addition, we determined if PHLPP promotes integrin ubiquitination since ubiquitination has been linked to lysosome-mediated degradation of integrin [21, 24]. Endogenous β4 and β1 integrin were immunoprecipitated from control and PHLPP overexpressing Panc-1 cells, and the ubiquitination levels of β4 and β1 integrin were increased in both PHLPP1β and PHLPP2 overexpressing cells (Figure 8D). Furthermore, since it has been shown that activation of PI3K/Akt signaling promotes integrin recycling to the plasma membrane by inhibiting GSK-3β [25], we examined if PHLPP-mediated downregulation of integrin can be rescued by inhibiting GSK-3 activity directly. Indeed, when treating cells with LiCl, a GSK-3 inhibitor, the expression of β1 and β4 integrin increased in both control and PHLPP overexpressing cells (Supplementary Figure S4). Taken together, our results suggest that PHLPP may downregulate β4 and β1 integrin expression by increasing the ubiquitination of these proteins, and inhibition of lysosome-mediated protein degradation rescues integrin expression in PHLPP overexpressing cells.

### Blocking integrin activity inhibits PHLPP-loss induced increase in cell motility

**Figure 9:**
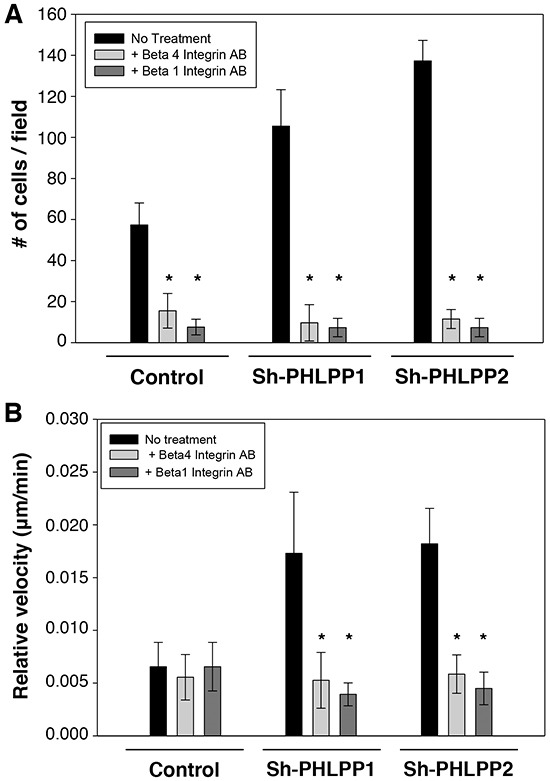
The PHLPP-mediated regulation of cell migration relies on its ability to control integrin expression **A.** Stable control and PHLPP knockdown ASPC-1 cells were subjected to Transwell migration analysis using HGF as the chemoattractant. To block integrin activation, ASPC-1 cell suspensions were incubated with blocking antibodies of β1 or β4 integrin in serum-free media containing 1% BSA for 20 minutes at 4°C. The cells were then seeded onto the collagen coated Transwells in the presence of the blocking antibodies and allowed to migrate for 4 hours at 37°C. Each experiment was done in duplicates and three independent experiments were averaged and expressed as mean ± SEM (* *p*<0.01 by two-sample *t*-tests compared to the control cells). **B.** Cells as treated as above with blocking antibodies were monitored for single cell motility using time-lapse imaging. The cells were allowed to migrate in response to HGF for 6 hours. The average velocities of 12 cells from each group are shown. Data represent mean ± SEM (*n* = 12 cells/line, * *p*<0.05 by two-sample *t*-tests).

To determine the functional impact of PHLPP-mediated inhibition of integrin expression, we examined the rate of cell migration in control and PHLPP knockdown ASPC-1 cells in the presence of blocking antibodies against β4 or β1 integrin. As shown in Figure 9A, incubating cells with either β4 or β1 integrin blocking antibodies significantly reduced cell migration in both control and PHLPP knockdown cells as measured by Transwell assays. Moreover, inhibiting integrin activation impaired cell motility at the single cell level as the relative velocity in the control and PHLPP knockdown cells became indistinguishable after the blocking antibody treatment (Figure 9B). These data suggested that increased integrin expression in PHLPP knockdown cells is responsible for increased cell migration.

## DISCUSSION

A number of recent studies have provided strong evidence suggesting that PHLPP serves as an important tumor suppressor in various cancer types [9, 10, 12, 15–17, 26]. For example, it has been shown that PHLPP is downregulated in colon cancer patients and decreased PHLPP expression promotes tumor growth as the result of increased activation of both PI3K/Akt and RAS/RAF oncogenic signaling [12, 13, 15]. However, the role of PHLPP in regulating cell migration has not been determined in pancreatic cancer. Here we show that PHLPP plays an important role in controlling cell motility. Specifically, overexpression of either PHLPP isoform decreases cell migration whereas knockdown of endogenous PHLPPs significantly enhances cell motility in pancreatic cancer cells. Moreover, the level of PHLPP expression negatively regulates the ability of pancreatic cancer cells to grow in 3D culture. This PHLPP-mediated inhibition of cell growth and migration is coincided with inhibition of both Akt and MEK/ERK activation. Mechanistically, PHLPP inhibits β1 and β4 integrin expression by increasing ubiquitination and lysosome-mediated degradation of integrin proteins, and blocking integrin activation reverses PHLPP-loss induced increase in cell migration. Consistent with a previously published study [14], we find that overexpression of PHLPP isoforms promote cell death but have little effect on proliferation in PDAC cells (data not shown). Taken together, our findings establish a novel link connecting PHLPP downregulation with enhanced integrin function in pancreatic cancer.

The cell surface delivery and endocytosis of integrins are dynamically regulated processes and fundamental for cell migration in wound healing and during cancer cell metastasis [27, 28]. Like many cell surface receptors, integrins are known to undergo endo/exocytic cycles, in which they are internalized, trafficked through endosome, and then recycled back to the plasma membrane [7, 28–30]. This recycle process occurs rather efficiently as the degradation rates of integrins are generally very slow [24, 31], however, ligand-bound integrins can be ubiquitinated and sorted to lysosome for degradation by the ESCRT pathway [24, 32]. Our findings that overexpression of PHLPP reduces the expression of β1 and β4 integrin and enhances their ubiquitination suggest that PHLPP may negatively control cell motility by inhibiting Akt activity to promote lysosome-dependent degradation of integrin. Since preventing lysosome-mediated degradation of integrins increases recycling of integrins to the plasma membrane and enhances integrin-mediated cancer cell migration and invasion [29, 30], PHLPP represents a novel regulator of cell motility by controlling the balance of integrin recycling and degradation in pancreatic cancer cells.

Previous studies have shown that the protein expression of both PHLPP isoforms are reduced in pancreatic cancer patient specimens compared to normal pancreases tissues [14]. We have interrogated the mRNA expression of PHLPP in several gene expression databases and found that the expression of PHLPP1 is significantly decreased in pancreatic tumor samples compared to normal samples whereas PHLPP2 mRNA expression is less affected (Supplementary Figure S5). Thus, loss of PHLPP protein expression in pancreatic cancer is likely mediated via a post-transcriptional or post-translational mechanism. Interestingly, recent studies have identified PHLPP2 as a bona fide target for a number of oncogenicmicroRNAs (miRNAs), including miR-372, miR-224 and miR-17~92, in difference cancer types [33–35]. In addition, single nucleotide variations in both PHLPP genes are rare (<1% based on COSMIC collections of somatic mutations). Future studies are needed to determine the mechanism leading to downregulation of PHLPP proteins.

Due to the late stage diagnosis and the aggressive nature of the carcinoma itself, PDAC patients have a very low survival rate [1, 4]. The treatment options for PDAC are few and surgical resection is nearly impossible for the average patient [36]. Understanding the molecular mechanism leading to EMT and progression of PDAC potentially has a long lasting benefit in creating new treatment strategies. In this study, we provide strong evidence supporting a tumor suppressor role of PHLPP in pancreatic cancer. Moreover, our finding that decreased PHLPP expression promotes EMT and cell motility through inhibiting lysosome degradation of integrin proteins identifies a novel mechanism by which PHLPP suppresses oncogenic signaling. Recent studies have demonstrated that upregulation of β4 integrin is associated with the EMT phenotype and poor prognosis in pancreatic cancer [37] and increased β1 integrin expression in tamoxifen-resistant breast cancer cells promotes EMT [38]. Future studies are needed to determine if PHLPP-loss induced EMT requires upregulation of integrin in PDAC cells. Collectively, our study highlights the importance of further exploring PHLPP as a possible diagnostic marker for targeted therapy in pancreatic cancer.

## EXPERIMENTAL PROCEDURES

### Cells and reagents

The following pancreatic ductal adenocarcinoma cell lines, including ASPC-1, Panc-1, and Suit-2, were kindly provided by Dr. Kathleen O'Connor (University of Kentucky). The Panc-1 cells used in this study were sorted for their high integrin expression as described previously [6]. Panc-1 cells were cultured in DMEM whereas ASPC-1 cells were cultured in RPMI-1640. All media were supplemented with 10% fetal bovine serum (FBS, Sigma-Aldrich) and 1% penicillin/streptomycin. The shRNAs used to knockdown human PHLPP1 or PHLPP2 gene and the retroviral expression plasmids, including pBabe-puro-HA-PHLPP1β and pBabe-puro-HA-PHLPP2, have been described previously [13, 15]. The stable PHLPP overexpressing cells were generated by infecting cells with retrovirus encoding HA-PHLPP1β or HA-PHLPP2 and selecting with puromycin (1 μg/ml).

The PHLPP1 and PHLPP2 antibodies were obtained from Proteintech and Bethyl Laboratories, respectively. The phospho-Akt (p-Akt for the Ser473 site), phospho-ERK1/2 (p-ERK for Thr202/Tyr204), phospho-MEK1/2 (p-MEK for Ser217/221), and E-cadherin antibodies were from Cell Signaling. The vimentin antibody was from BD Biosciences, the γ tubulin antibody was from Sigma-Aldrich, and the ubiquitin antibody was from Santa Cruz Biotechnology. The following integrin antibodies were used in this study: for Western blotting, anti-β1 rabbit mAb (D2E5, Cell Signaling) and anti-β4 mouse mAb (clone 7, BD Biosciences); and for blocking ECM binding and immunoprecipitation, anti-β1 mouse mAb (clone 6S6, Millipore) and anti-β4 mouse mAb (clone ASC-8, Millipore). The following chemicals, including PI3K inhibitor LY290042 and Akt inhibitor VIII, were purchased from EMD/CalBiochem. The MEK1/2 inhibitor PD0325901 was purchased from Selleck Chemicals, and lysosome inhibitor chloroquine was from Sigma-Aldrich.

### Immunoblotting

Cultured cells were harvested and lysed in Lysis Buffer (50 mM Na_2_HPO_4_, 1 mM sodium pyrophosphate, 20 mM NaF, 2 mM EDTA, 2 mM EGTA, 1% Triton X-100, 1 mM DTT, 200 mM benzamidine, 40 mg ml^−1^ leupeptin, 200 mM PMSF) and the detergent-solublized cell lysates were obtained after centrifugation for 5 minutes at 16,000 g at 4°C. Equal amounts of cell lysates as determined by Bradford assays were resolved by SDS-PAGE and subjected to immunoblotting analysis. The density of ECL signals was obtained and quantified using a FluoChem digital imaging system (Alpha Innotech).

### Transwell migration assays

Serum starved pancreatic cancer cells were seeded into the upper chamber of Transwell inserts with an 8-mm pore size membrane (Corning). For migration assays, the bottom of the inserts were coated with collagen (15 mg/ml) or laminin (25 μg/ml), and the cells were allowed to migrate towards media containing either HGF (20 ng/ml) or IGF (20 ng/ml) in the lower chamber for 2-5 hours. At the end of the incubation period, Transwell inserts were fixed in methanol and stained with 0.5% crystal violet. The numbers of cells that were migrated to the lower chamber of the Transwell were counted using an inverted microscope at 20X magnification.

### Time-lapse live cell imaging and analysis

Stable control and PHLPP overexpressing Panc-1 cells and PHLPP knockdown ASPC-1 cells were serum starved for 2 hours and seeded as single cells onto collagen (15 mg/ml) or laminin (25 mg/ml) coated glass bottom culture dishes (MatTek). The migration of live cells was recorded in media containing HGF (20 ng/ml) using Nikon BioStation IM equipped with CO_2_ incubation chamber. Time-lapse phase images were taken either every 5 or 10 minutes for 6 hours with a 20X objective. The movement of cells was tracked and analyzed using Nikon Element AR software.

### 3D cell culture

Single cell suspension of pancreatic cancer cells was prepared in serum free media and embedded into collagen/Matrigel (1:1) mixture. The cells were overlaid with 2% Matrigel in 2% FBS containing media. The cells were allowed to grow into cyst-like structures in the 3D matrix for 1-2 weeks. The size and morphology of the cysts were examined by phase-contrast microscopy using a Nikon Ti-E inverted microscope. For immunofluorescence staining, the cysts were fixed in 4% paraformaldehyde and permeabilized using 1% Triton X-100 in PBS. Actin was stained using Alexa 488-conjugated phalloidin while the nuclei of the cells were stained with DAPI-containing mounting medium. Cells were visualized using an Olympus FlowView FV1000 confocal laser-scanning microscope.

### Real-time PCR

Total RNA was isolated with RNeasy kit (Qiagen) from different stable pancreatic cancer cell lines. Equal amounts of RNA were used as templates for the synthesis of cDNA using High Capacity cDNA Reverse Transcription kit (Applied Biosysems). Real-time PCR reaction was performed using the StepOne Real-Time PCR system (Applied Biosysems) with β1- and β4-specific probes. All values were normalized to the level of β-actin.

### Statistical analysis

Normalized microarray and patient clinical data from two pancreatic cancer studies [39, 40] were downloaded from the Oncomine database. The Badea et al data [40] contain matched tumor and normal samples from 36 pancreatic cancer patients. The TCGA data [39] contain tumor samples from 65 pancreatic cancer patients and normal samples from 65 patients, within which 64 patients have both tumor and normal samples. Expression levels of multiple probes mapping to the same gene were averaged. Gene expressions from replicated tumor or normal samples for the same patient were also averaged. The paired *t*-test or linear mixed model was used to compare PHLPP1 and PHLPP2 expressions between tumor and normal samples.

## SUPPLEMENTARY FIGURES


