# Supplementary material for: PHLPP negatively regulates cell motility through inhibition of Akt activity and integrin expression in pancreatic cancer cells
Source: Oncotarget. 2016 Jan 8;7(7):7801–15. doi: 10.18632/oncotarget.6848 (PMC4884955; doi:10.18632/oncotarget.6848)
Supplement: Supplementary file 1 [file oncotarget-07-7801-s001.pdf]

SUPPLEMENTARY FIGURES

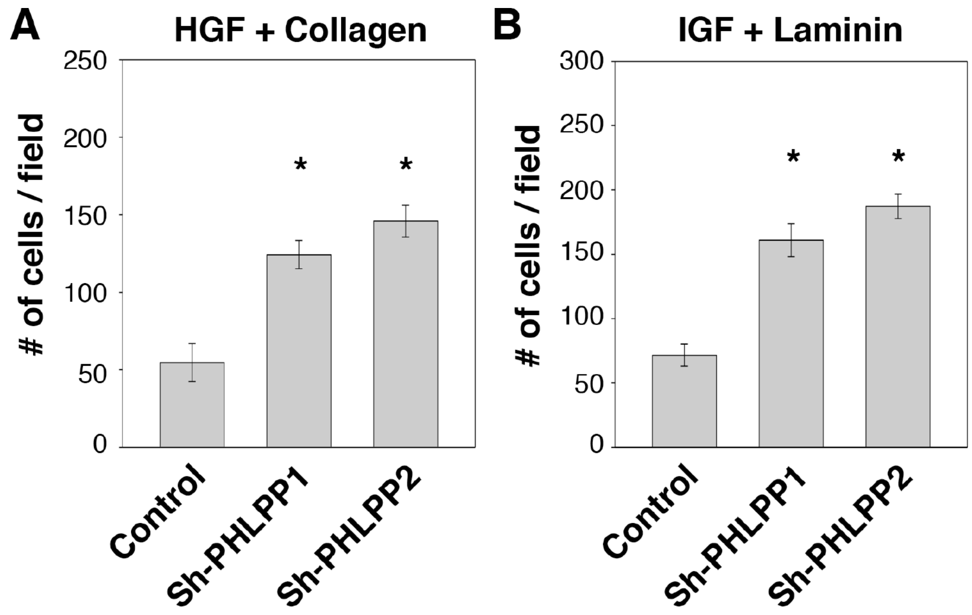

Supplementary Figure S1: Knockdown of PHLPP expression promotes cell migration. Stable control and PHLPP knockdown ASPC-1 cells were subjected to Transwell migration assays using either HGF and collagen **A.** or IGF-1 (20 ng/ml) and laminin **B.** as chemoattractants. Each experiment was done in duplicates and three independent experiments were averaged and expressed as mean ± SD (* p<0.05 by two-sample t-tests compared to the control cells).

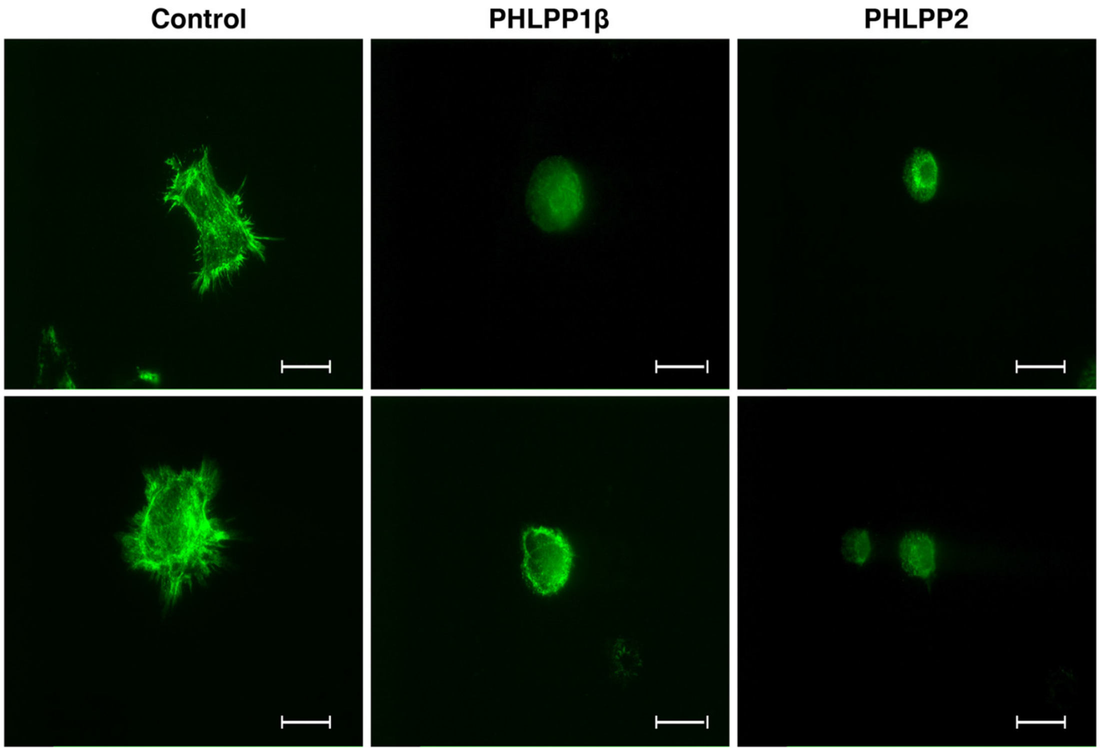

Supplementary Figure S2: PHLPP affects actin cytoskeleton dynamic in pancreatic cancer cells. Stable control and PHLPP overexpressing Panc-1 cells were seeded onto collagen coated cover slips in serum-free media. After 20 minutes of incubation at 37°C the cells were fixed with 4% paraformaldehyde, blocked with anti-goat serum and stained with Alexa488-phalloidin. TIRF images were from the basolateral membrane of the cells and two representative images are shown for each cell line (scale bars, 100 µm).

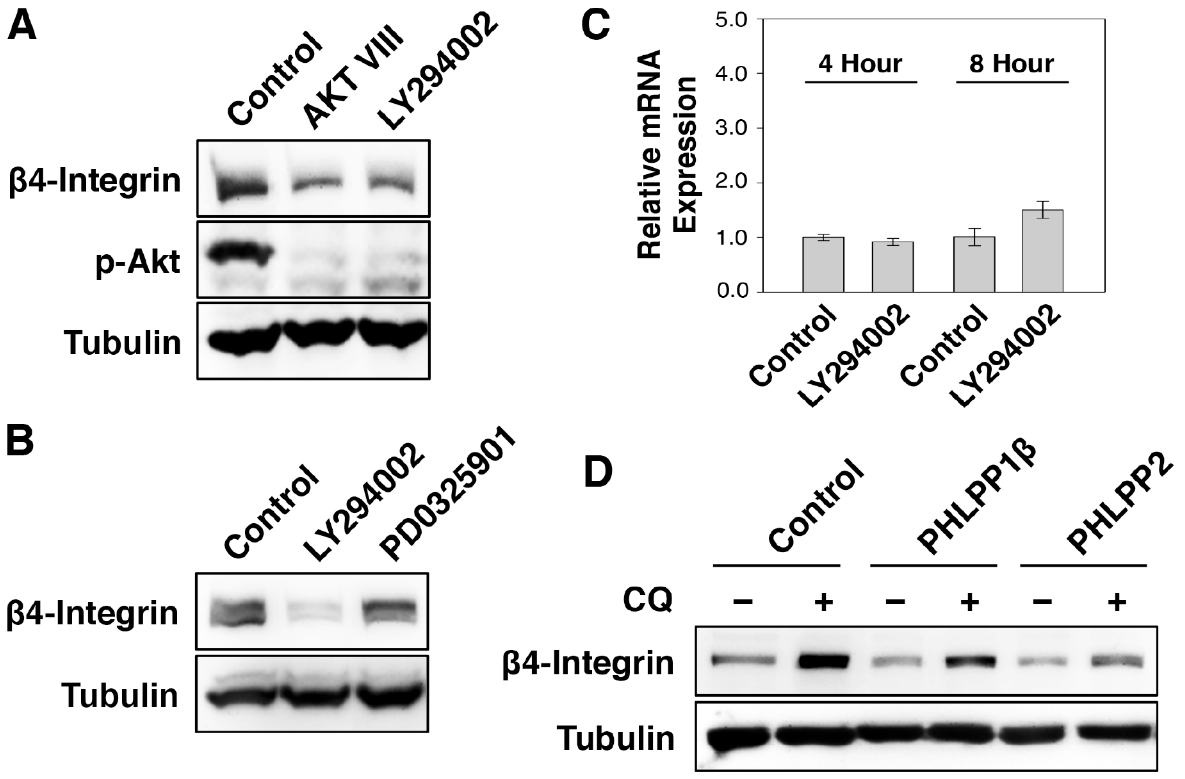

Supplementary Figure S3: The effect of inhibiting PI3K/Akt and MEK/ERK signaling on the expression of integrins in pancreatic cancer cells. A. ASPC-1 cells were incubated with DMSO, AKT inhibitor (AKT VIII, 0.5 µM), or LY294002 (20 µM) for 8 hours. Cell lysates were prepared and analyzed for the expression of β4 integrin, p-AKT, and tubulin using immunoblotting. **B.** ASPC-1 cells were treated with DMSO, LY294002 (20 µM) or PD0325901 (10 nM) for 16 hours. Cell lysates were prepared and analyzed for the expression of β4 integrin and tubulin using immunoblotting. **C.** Panc-1 cells were treated with LY204002 (20 µM) for 4 or 8 hours and total RNAs were extracted. Real-time PCR analysis was performed using probes specific for human β4 integrin gene. Each experimental point was done in triplicates, and the graphs represent the mean ± SD (*n*=3). *D.* Stable control and PHLPP overexpressing Panc-1 cells were treated with Chloroquine (CQ, 20 µM) for 4 hours. Cell lysates were prepared and analyzed by immunoblotting for β4 integrin expression.

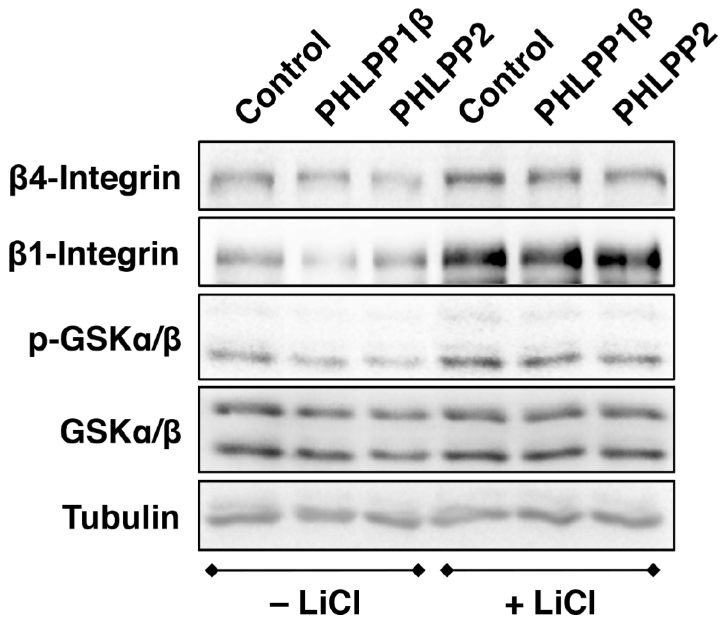

Supplementary Figure S4: Inhibition of GSK3 activity increased integrin expression. Stable control and PHLPP overexpressing Panc-1 cells were treated with GSK3 inhibitor LiCl (10 mM) for 24 hours. Cell lysates were prepared and analyzed for the expression of β4 and β1 integrin using immunoblotting. Note that overexpression of either PHLPP isoform decreased phosphorylation of GSK3 suggesting increased GSK3 activity.

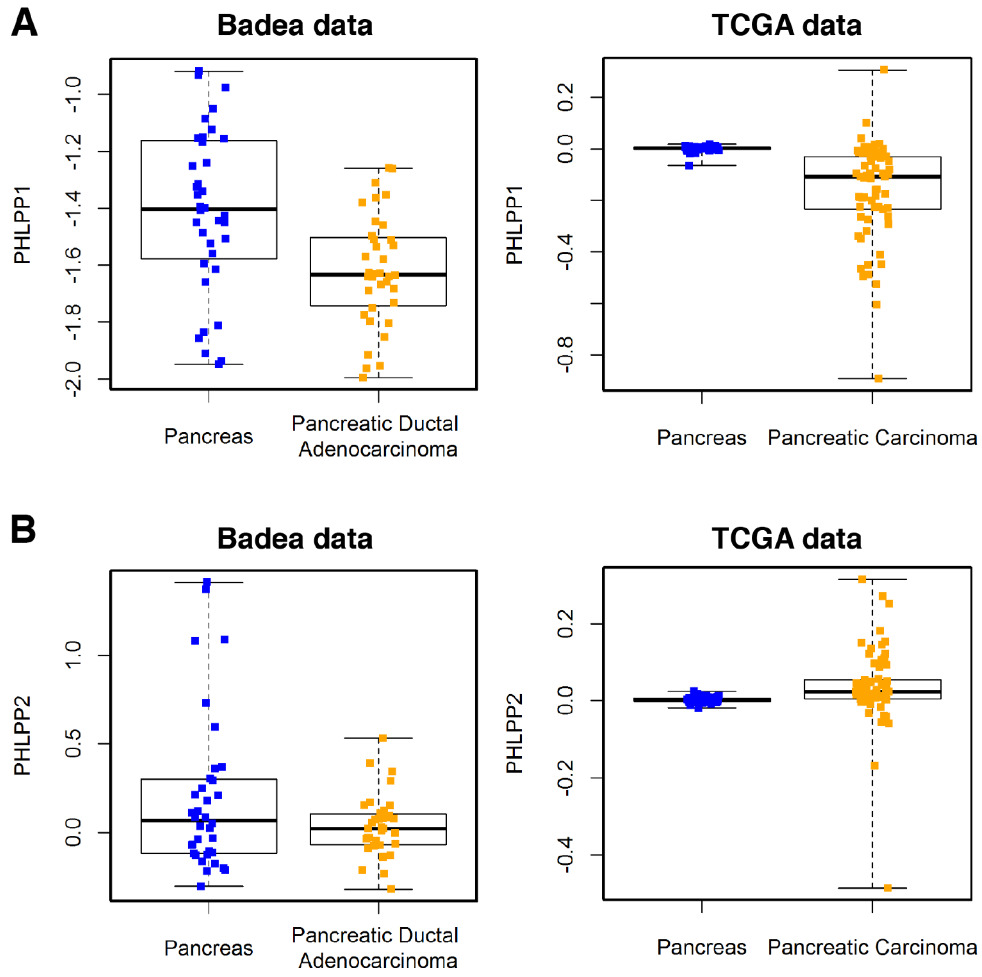

Supplementary Figure S5: The expression of PHLPP1 and PHLPP2 gene in pancreatic cancer patient samples. Expression of PHLPP1 was significantly lower in pancreatic tumor samples compared to normal samples in the data from Badea et al. [1] ($P<0.001$ based on a paired *t*-test) and in the data from TCGA [2] ($P<0.001$ based on a linear mixed model). The association between PHLPP2 expression and pancreatic cancer was less clear. PHLPP2 was upregulated in tumor samples based on the TCGA data ($P = 0.010$) but downregulated based on the Badea et al. data ($P = 0.04$).

REFERENCES

1. Badea L, Herlea V, Dima SO, Dumitrascu T, Popescu I. Combined gene expression analysis of whole-tissue and microdissected pancreatic ductal adenocarcinoma identifies genesspecifically overexpressed in tumor epithelia. Hepatogastroenterology. 2008; 55:2016–2027.
2. TCGAThe Cancer Genome Atlas. http://tcga-datancinihgov/tcga/.
